# Combined quantitation of *HMGA2* mRNA, microRNAs, and mitochondrial-DNA content enables the identification and typing of thyroid tumors in fine-needle aspiration smears

**DOI:** 10.1186/s12885-019-6154-7

**Published:** 2019-10-28

**Authors:** Sergei E. Titov, Mikhail K. Ivanov, Pavel S. Demenkov, Gevork A. Katanyan, Eugenia S. Kozorezova, Anastasia V. Malek, Yulia A. Veryaskina, Igor F. Zhimulev

**Affiliations:** 1 Institute of Molecular and Cellular Biology, Novosibirsk, 630090 Russia; 2 AO Vector-Best, Koltsovo, 630559 Russia; 3 Institute of Cytology and Genetics, Novosibirsk, 630090 Russia; 4 Novosibirsk State University, Novosibirsk, 630090 Russia; 5 Regional Clinical Hospital No. 2, Krasnodar, 350012 Russia; 6 Siberian District Medical Center of Federal Medical and Biological Agency, Novosibirsk, 630007 Russia; 7 N.N. Petrov National Medical Research Center of Oncology, St. Petersburg, 197758 Russia

**Keywords:** Thyroid carcinoma, preoperative diagnosis, molecular markers, HMGA2, microRNA

## Abstract

**Background:**

Analysis of molecular markers in addition to cytological analysis of fine-needle aspiration (FNA) samples is a promising way to improve the preoperative diagnosis of thyroid nodules. Nonetheless, in clinical practice, applications of existing diagnostic solutions based on the detection of somatic mutations or analysis of gene expression are limited by their high cost and difficulties with clinical interpretation.

The aim of our work was to develop an algorithm for the differential diagnosis of thyroid nodules on the basis of a small set of molecular markers analyzed by real-time PCR.

**Methods:**

A total of 494 preoperative FNA samples of thyroid goiters and tumors from 232 patients with known histological reports were analyzed: goiter, 105 samples (50 patients); follicular adenoma, 101 (48); follicular carcinoma, 43 (28); Hürthle cell carcinoma, 25 (11); papillary carcinoma, 121 (56); follicular variant of papillary carcinoma, 80 (32); and medullary carcinoma, 19 (12). Total nucleic acids extracted from dried FNA smears were analyzed for five somatic point mutations and two translocations typical of thyroid tumors as well as for relative concentrations of *HMGA2* mRNA and 13 microRNAs and the ratio of mitochondrial to nuclear DNA by real-time PCR. A decision tree–based algorithm was built to discriminate benign and malignant tumors and to type the thyroid cancer. Leave-p-out cross-validation with five partitions was performed to estimate prediction quality. A comparison of two independent samples by quantitative traits was carried out via the Mann–Whitney *U* test.

**Results:**

A minimum set of markers was selected (levels of *HMGA2* mRNA and miR-375, − 221, and -146b in combination with the mitochondrial-to-nuclear DNA ratio) and yielded highly accurate discrimination (sensitivity = 0.97; positive predictive value = 0.98) between goiters with benign tumors and malignant tumors and accurate typing of papillary, medullary, and Hürthle cell carcinomas. The results support an alternative classification of follicular tumors, which differs from the histological one.

**Conclusions:**

The study shows the feasibility of the preoperative differential diagnosis of thyroid nodules using a panel of several molecular markers by a simple PCR-based method. Combining markers of different types increases the accuracy of classification.

## Background

Thyroid nodules are the commonest pathology of the endocrine system. According to different estimates [1, 2], ~5–15% of them are malignant and require a surgical intervention. The decision about such an intervention is based on the cytological examination of preparations obtained by fine-needle aspiration cytology (FNAC). This procedure requires a highly experienced specialist, but even if the FNA procedure is performed with the highest accuracy, diagnostic uncertainty is not eliminated in ~30% of the cases because the cytological characteristics are not sufficient to discriminate between benign and malignant follicular tumors [3]. In these cases, the final diagnosis can be made only after surgical resection and a histological examination of the surgical specimens. As a consequence, some of the interventions appear to be too extensive or even unnecessary. On the other hand, 15–40% of FNAC results include atypia of undetermined significance and nondiagnostic or unsatisfactory material that typically requires a repeat biopsy [3]. This situation hinders timely treatment of some malignant tumors. Thus, it is necessary to improve the methods of preoperative diagnosis of thyroid tumors.

There is an effort to combine the results of FNAC with those of other diagnostic methods, such as ultrasonography, immunocytochemistry, and detection of molecular markers. These studies have led to the development of diagnostic solutions based on different types of molecular markers (e.g., somatic mutations and translocations and expression levels of protein-coding genes and microRNAs [miRNAs]) [4–7]. Testing of these solutions and accumulated evidence show that the diagnostic potential of various molecular markers is determined by their characteristic limitations. This is due to both biological and technical issues.

Diagnostic solutions based on the detection of somatic mutations (ThyroSeq®, CBLPath), expression profiling of a variety of protein-coding genes (Afirma® Gene Expression Classifier and Genomic Sequencing Classifier, Veracyte), miRNA profiling (Rosetta GXReveal, Rosetta Genomics), or a combined analysis of somatic mutations and miRNAs (ThyGenX/ThyraMIR, Interpace Diagnostics) have already been used in practice for preoperative diagnosis. Despite the reported high negative predictive value (NPV; from 91% for Rosetta GXReveal to 96% for ThyroSeq 2.0), the existing solutions have lower positive predictive value (PPV; 40–80% for various tests) [7]. This state of affairs along with the high cost of analysis ($3000–3500 per patient) prevents their widespread use in clinical practice. It is possible that testing different types of molecular markers in parallel, together with FNAC, will improve overall accuracy of the analysis. Nonetheless, parallel use of the above approaches for testing the same nodule is problematic because the analysis of various types of markers requires different sample preparation procedures and different instrumentation.

In this regard, the aim of our work was to select a small set of molecular markers for preoperative detection of cancer and for typing thyroid tumors with high throughput and acceptable accuracy, by means of the same cytological specimen for each patient, the same simple method for isolating nucleic acids, and the same assay, real-time PCR. Five types of molecular markers were chosen: somatic point mutations in genes *BRAF*, *HRAS*, and *NRAS* and translocations *RET-PTC1* and *PAX8-PPARγ*, which are the most common in thyroid carcinomas; a normalized concentration of *HMGA2* mRNA; normalized levels of several miRNAs as well as the ratio of mitochondrial to nuclear DNA (mtDNA/nDNA). The last marker, which is not employed in existing diagnostic solutions, was added here to detect Hürthle cells in the clinical specimen.

## Methods

### Clinical material

This material included 501 cytological specimens obtained by FNA biopsy from 239 patients. The specimens were provided by the following healthcare institutions: N.N. Petrov National Medical Research Center of Oncology (St. Petersburg, Russia), 189 specimens (118 patients); Regional Clinical Hospital No. 2 (Krasnodar, Russia), 203 specimens (78 patients); and the Siberian District Medical Center of the Federal Medical and Biological Agency (Novosibirsk, Russia), 109 specimens (43 patients). All three hospitals are located in mildly iodine-deficient areas. The specimens were collected during 2016–2017. Cytology smears were air dried without fixation and then stained with hematoxylin and eosin. For each specimen, a pathology report was available. Histological examination was carried out by the staff pathologists of the respective institutions. During preoperative examination, several biopsies are usually taken from a single nodule; these samples in some cases may differ in a molecular-marker profile. Such heterogeneity may contribute to discrepancies between the results of the proposed preoperative molecular classifier and the histological diagnosis. To identify and take into account the cases of such discrepancies, for each patient for whom several FNAC slides were available (obtained from one nodule), we intentionally selected those that were quite different in at least one molecular marker. This approach was possible also because we used the decision tree method for prediction. It is not a statistical method; it actually involves a fairly simple algorithm of greedily finding the best splits in the data so that entropy is maximized in the split as measured using the outcome. This method does not require the assumption of independence because it does not affect the prediction [8].

When designing our study, we focused on evaluating the accuracy of the test results and therefore the selected specimens belonged only to three of the six Bethesda categories, namely, categories II, IV, and VI. Sixty-four specimens were collected from males (27 patients) with median age 53.9 ± 13.6 years (mean ± SD) and 437 specimens from females (212 patients) with median age 52.7 ± 14.8. The distribution of samples was as follows: goiter (colloid [non-neoplastic] nodules and Hashimoto’s thyroiditis), 107 specimens (50 patients); follicular thyroid adenoma (FTA), 103 (48 patients); follicular thyroid carcinoma (FTC), 44 (28 patients); Hürthle cell carcinoma (HCC), 25 (11 patients); papillary thyroid carcinoma (PTC), 121 (56 patients); follicular variant of papillary thyroid carcinoma (FVPTC, all invasive), 80 (32 patients); medullary thyroid carcinoma (MTC), 19 (12 patients); and anaplastic thyroid carcinoma, two (two patients). The specimens of anaplastic carcinoma were not used for developing the classifier owing to their insufficient quantity. Five specimens (one diagnosed by the pathology report as FTC, two as FTA, and two as goiter) were later excluded from the analysis because of the V600E mutation in the *BRAF* gene detected therein, which is specific to PTC. We assumed a possible error in the pathology reports on these five specimens (because mutant DNA in these tissue samples constituted approximately 10% to 40%) but did not have a chance for any verification. Thus, the ultimate sample for statistical analysis included 494 selected specimens from 232 patients.

### Nucleic-acid extraction

Total nucleic acids were extracted as described in ref. [9] with a slight modification, i.e., the dried cytological preparation was washed into a tube with three 200 μl portions of guanidine lysis buffer. The concentration of the isolated total RNA was measured on a NanoDrop 2000C spectrophotometer (Thermo Scientific, USA). Total-RNA concentrations were in the range of 1.2–92.6 ng/μl (mean 30.1 ng/μl). Samples were excluded from the analysis if the total-RNA concentration was below 5 ng/μl (2% of all the samples under study).

### Oligonucleotide primers and probes

All oligonucleotides were synthesized at AO Vector-Best (Russia). The oligonucleotides were selected using the PrimerQuest online service [10]. Sequences of primers and fluorescently labeled probes are presented in Additional file 1: Table S1.

### Selecting the set of molecular markers

The initial selection of a set of somatic mutations and translocations as well as mRNAs for analysis was based on the data available from relevant literature. The sets of oligonucleotides were chosen so that mRNA detection would be possible without prior removal of genomic DNA. The set of mutations and translocations included the V600E mutation in the *BRAF* gene, the Q61R mutation in *HRAS*, mutations Q61K, Q61R, and Q61L in *NRAS* [11–13], the M918T mutation in *RET* [14], and translocations *RET-PTC1*, *RET-PTC3*, and *PAX8-PPARγ* [11, 13]. The set of mRNAs included *HMGA2* [15, 16], *Ki-67* [17], and *IMP3* [18, 19]. For all the above markers, except for *PAX8-PPARγ*, oligonucleotide sets were designed and tested on a small subsample of the clinical material. The *RET* M918T mutation, the *RET-PTC3* translocation, and *Ki-67* and *IMP3* mRNAs were subsequently excluded from the analysis because they were insufficiently informative.

The choice of the miRNA set was based on the analysis of published data and our original preliminary results. A list of 13 miRNAs – malignancy markers – was compiled (miR-144-5p, -145-5p, -155-5p, -146b-5p, -183-5p, -199b-5p, -221-3p, -223-3p, -31-5p, -375, -451a, -551b-3p, and -7-5p), for which a set of three reference miRNAs was selected too (miR-197-3p, -23a-3p, and -29b-3p) [9, 20–25].

The ratio between the copy numbers of mtDNA and nDNA was added as an indicator of Hürthle cells in a clinical sample because, according to the literature [26], the mtDNA content of these cells is significantly higher.

### MiRNA detection and quantitation

The detection of the 16 miRNAs was performed on goiters and all the types of tumors. Mature miRNAs were identified by stem-loop real-time PCR [27]. For each miRNA, a reverse transcription (RT) reaction was carried out separately with subsequent real-time PCR. The reverse-transcription reaction and real-time PCR were conducted as described in ref. [9]. Each specimen was analyzed once. The miRNA content was normalized to the geometric mean of the amounts of the three reference miRNAs by the 2^-ΔCq^ method [28].

### Identification of somatic mutations and translocations

All the specimens were analyzed for the following somatic mutations: *BRAF* V600E, *HRAS* Q61R, *NRAS* Q61K, *NRAS* Q61R, and *NRAS* Q61L and for the *RET-PTC1* translocation. Only samples derived from follicular tumors were analyzed for the *PAX8-PPARγ* translocation. Real-time PCR and real-time RT-PCR were carried out on a CFX96 thermocycler (Bio-Rad Laboratories, USA) with lyophilized ready-to-use reaction mixtures Master-mix PCR and Master-mix RT-PCR (AO Vector-Best, Russia). The standard primer concentration in all the reactions was 0.5 μM, whereas the concentration of the fluorescently labeled probe was 0.25 μM.

The detection of somatic mutations was conducted by allele-specific PCR. The thermal cycling conditions were as follows: preheating for 2 min at 95°C and then 60 cycles of denaturation for 10 sec at 94°C with annealing and elongation for 15 sec at 60°C. Sensitivity of mutant-allele detection was determined on control samples with known concentrations corresponding to a mutant allele and wild-type allele. For detection of the *BRAF* mutation, the selectivity was 0.5% of the mutant allele, and for the identification of *HRAS* and *NRAS* mutations, it was 2%.

The detection of the *RET-PTC1* translocation was performed by single-tube real-time RT-PCR. The following RT-PCR program was employed: incubation for 30 min at 45°C, preheating for 2 min at 95°C, and then 50 cycles of denaturation for 10 sec at 94°C with annealing and elongation for 20 sec at 60°C.

The detection of the *PAX8-PPARγ* translocation was conducted using the PAX-PPARG Fusion GE Assays Reagent Kit (P7P2, P8P2, P9P2, and P10P2; Thermo Fisher, USA).

### Quantification of *HMGA2* mRNA

The relative concentration of *HMGA2* mRNA was estimated by real-time RT-PCR, where *PGK1* (phosphoglycerate kinase 1) mRNA served for normalization. The relative expression level was calculated by the 2^-ΔCq^ method.

### Determination of the mtDNA/nDNA ratio of copy numbers

Detection of specific sites in mtDNA and nDNA was performed independently by real-time PCR. The thermal cycling conditions were as follows: preheating for 2 min at 95°C then 50 cycles of denaturation for 10 sec at 94°C with annealing and elongation for 20 sec at 60°C. The ratio was determined by the 2^-ΔCq^ method.

### Classification

The classification of thyroid cytological preparations on the basis of the molecular markers was performed in the TANAGRA software [29] via the C4.5 decision tree algorithm [30]. Assessment of classification quality was made by leave-p-out cross-validation with five partitions (see the Results section for the principles of grouping of the specimens).

### Statistical analysis

This analysis was carried out using software package Statistica 9.1 (StatSoft Inc., USA). Two independent samples were compared by quantitative traits via the Mann–Whitney *U* test.

## Results

### The proposed analysis of somatic mutations and translocations has different specificity and sensitivity levels for different types of tumors and goiters

**Fig. 1:**
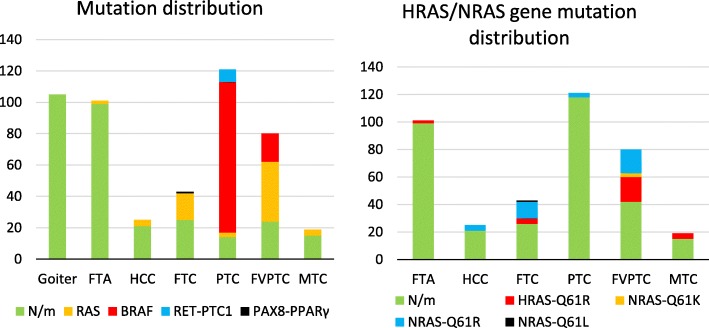
Distribution of mutations among goiters and various tumor types. The number of samples is presented. Left: The numbers of various types of detected mutations; right: the numbers of various types of mutations detected in genes *HRAS* and *NRAS*. N/m denotes the samples where no mutations were detected

As expected, the *BRAF* V600E mutation and *RET-PTC1* translocation were detected in the samples of papillary carcinoma only, whereas the *PAX8-PPARγ* translocation was found only in follicular carcinomas. The *RAS* mutations did not show such specificity to the tumor type and were found in follicular, papillary, and medullary carcinomas and HCCs. In this study, these mutations were most often detected in FVPTCs (48%) and FTCs (40%) and less frequently in MTCs (21%) and HCCs (16%; Fig. 1, left). When the follicular neoplasms (FNs) were categorized into benign and malignant according to the presence or absence of *RAS* mutations, sensitivity was 0.40, specificity 0.98, PPV 0.89, and NPV 0.79. The prevalence of particular mutations in the *RAS* genes appeared to vary somewhat depending on the tumor type (Fig. 1, right). In most cases, the *BRAF*, *NRAS*, and *HRAS* mutations and the *RET-PTC1* translocation were mutually exclusive. An exception was a single case of classic PTC where a mutation in codon 61 of the *NRAS* gene and the *RET-PTC1* translocation were both detected. None of the studied mutations was detected in goiters.

### *HMGA2* overexpression is a marker of malignancy

**Fig. 2:**
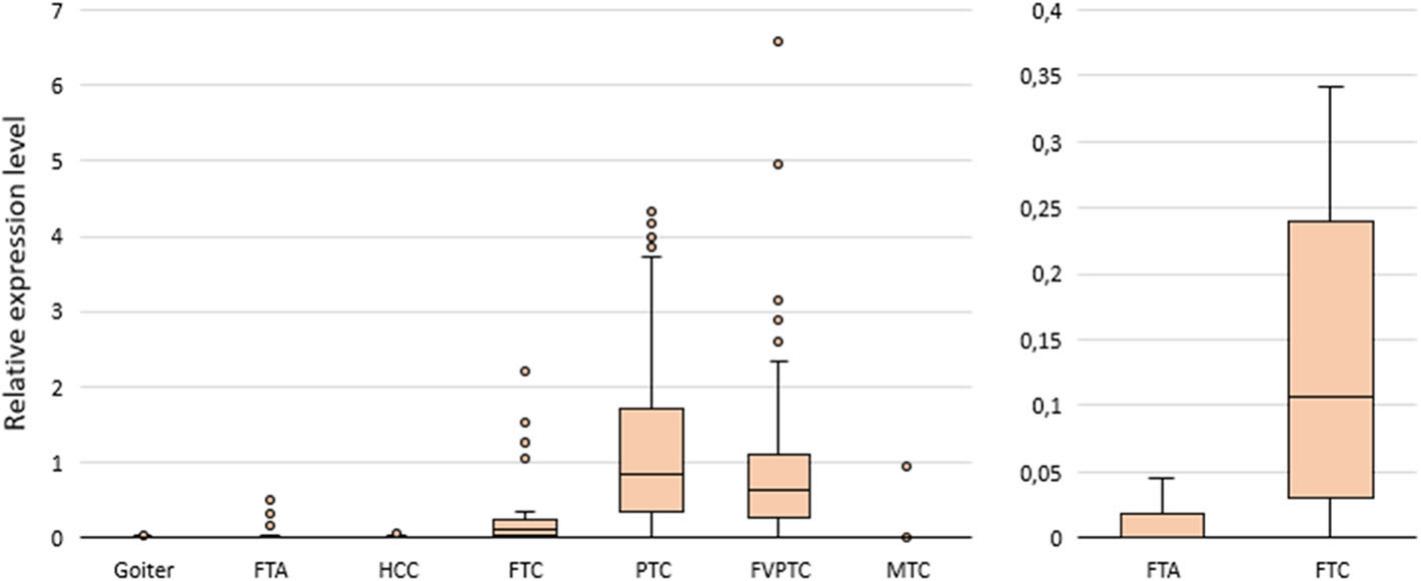
*HMGA2* mRNA expression in goiters and thyroid tumors. Left: The relative level of *HMGA2* mRNA in goiters and different tumor types. Right: The level of *HMGA2* mRNA expression in follicular cancers (*n* = 43) and follicular adenomas (corresponds to Fig. 2 [left], excluding the outliers). The figure presents the median value, upper and lower quartiles, nonoutlier range, and outliers (circles)

Levels of *HMGA2* mRNA in goiters and various tumor types are presented in Fig. 2, and the P values for different tumor types compared pairwise are listed in Table 1.

**Table 1:** *P* values for pairwise comparisons of *HMGA2* gene expression between goiters and various tumor types

	Goiter	FTA	FTC	HCC	PTC	FVPTC	MTC
Goiter		0.0151	1.7 × 10^− 12^	0.5524	0	1.9 × 10^− 30^	0.1727
FTA			2.6 × 10^−13^	0.3081	0	4.5 × 10^−30^	0.5859
FTC				2.9 × 10^−7^	1.4 × 10^−9^	9.5 × 10^−8^	6.6 × 10^−6^
HCC					4.7 × 10^−14^	1.4 × 10^−13^	0.4822
PTC						0.0648	1.8 × 10^−10^
FVPTC							5.6 × 10^− 10^

*HMGA2* mRNA overexpression was mostly detected in classic PTC and FVPTC and less frequently in FTC. In PTCs, the *HMGA2* mRNA level was ~200-fold higher on average compared with that in goiters. There were also several samples with overexpressed *HMGA2* mRNA among FTAs (*n* = 5; 5%). Two specimens of anaplastic carcinoma also featured an increased level of *HMGA2* mRNA. On the other hand, a high *HMGA2* mRNA level was detected only in one of 19 medullary carcinomas derived from parafollicular cells (С-cells) and was not detected among HCCs.

In comparison with other markers, the *HMGA2* expression level was the best stand-alone marker for discrimination among goiters, benign tumors, and malignant tumors. The area under the receiver-operating characteristic (ROC) curve (AUC) for the discrimination of goiters, benign tumors, and malignant tumors when the histological analysis served as a reference method was 0.884 ± 0.015, and when we excluded HCC and MTC, AUC was 0.959 ± 0.009, but in the analysis of FNs, it was only 0.861 ± 0.037. For the discrimination of malignant and benign tumors from the FN group on the basis of the *HMGA2* expression level using the cutoff value selected by the ROC analysis, sensitivity was 0.72, specificity 0.91, PPV 0.78, and NPV 0.88. See also Additional file 2: Table S2, where the data on goiters and each tumor type are provided.

### The mtDNA/nDNA ratio is significantly higher in Hürthle cell carcinomas

**Fig. 3:**
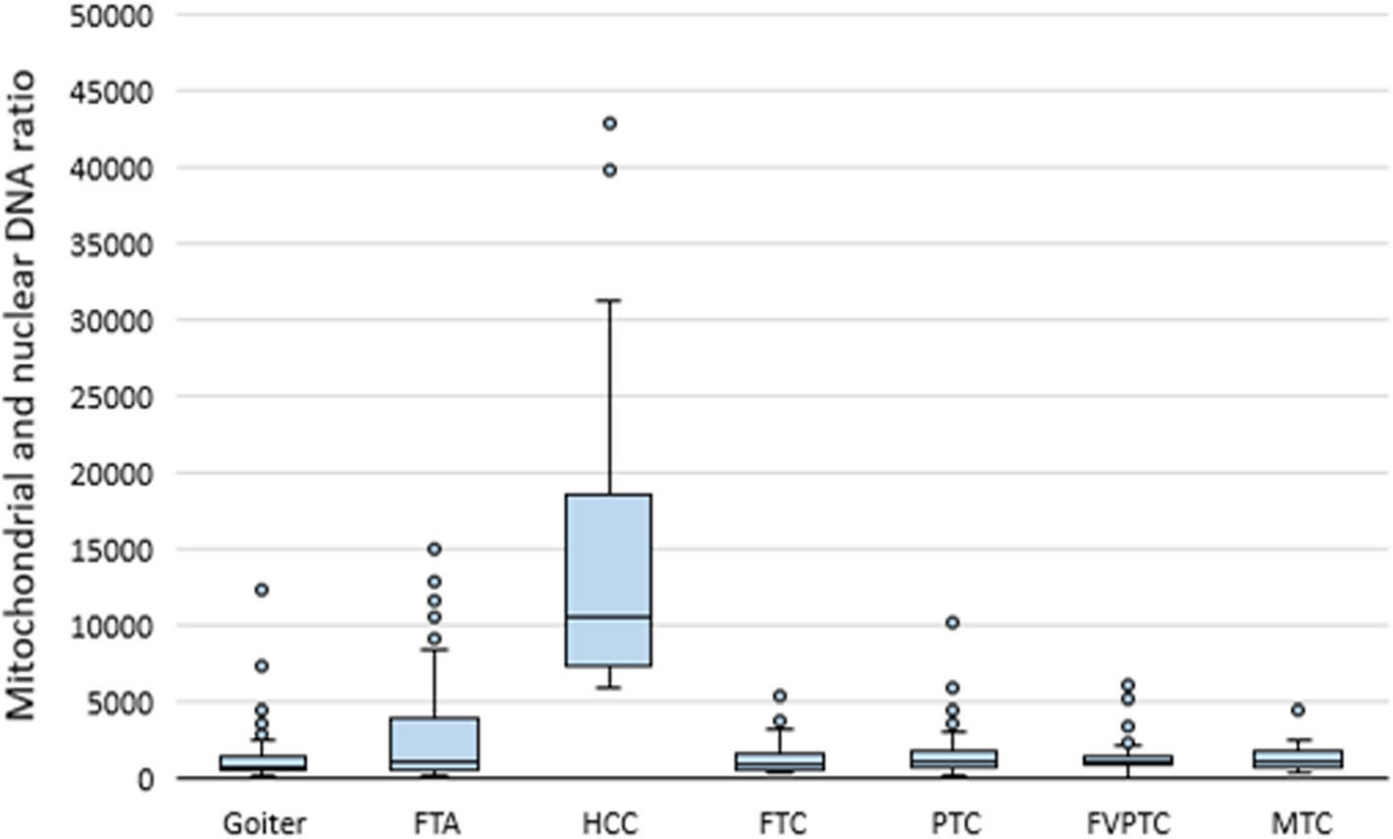
The mtDNA/nDNA ratio in goiters and various tumor types. The figure presents the median value, upper and lower quartiles, nonoutlier range, and outliers (circles)

This ratio was significantly higher in HCCs than in all other groups (*P* value 1.96 × 10^-8^ to 7.31 × 10^-15^). Nevertheless, several samples with a high mtDNA/nDNA ratio were identified among the goiters, PTCs, and FTAs (Fig. 3).

### The levels of selected miRNAs differ significantly between goiters and various tumor types, but none of them is a “universal” marker of malignancy

**Fig. 4:**
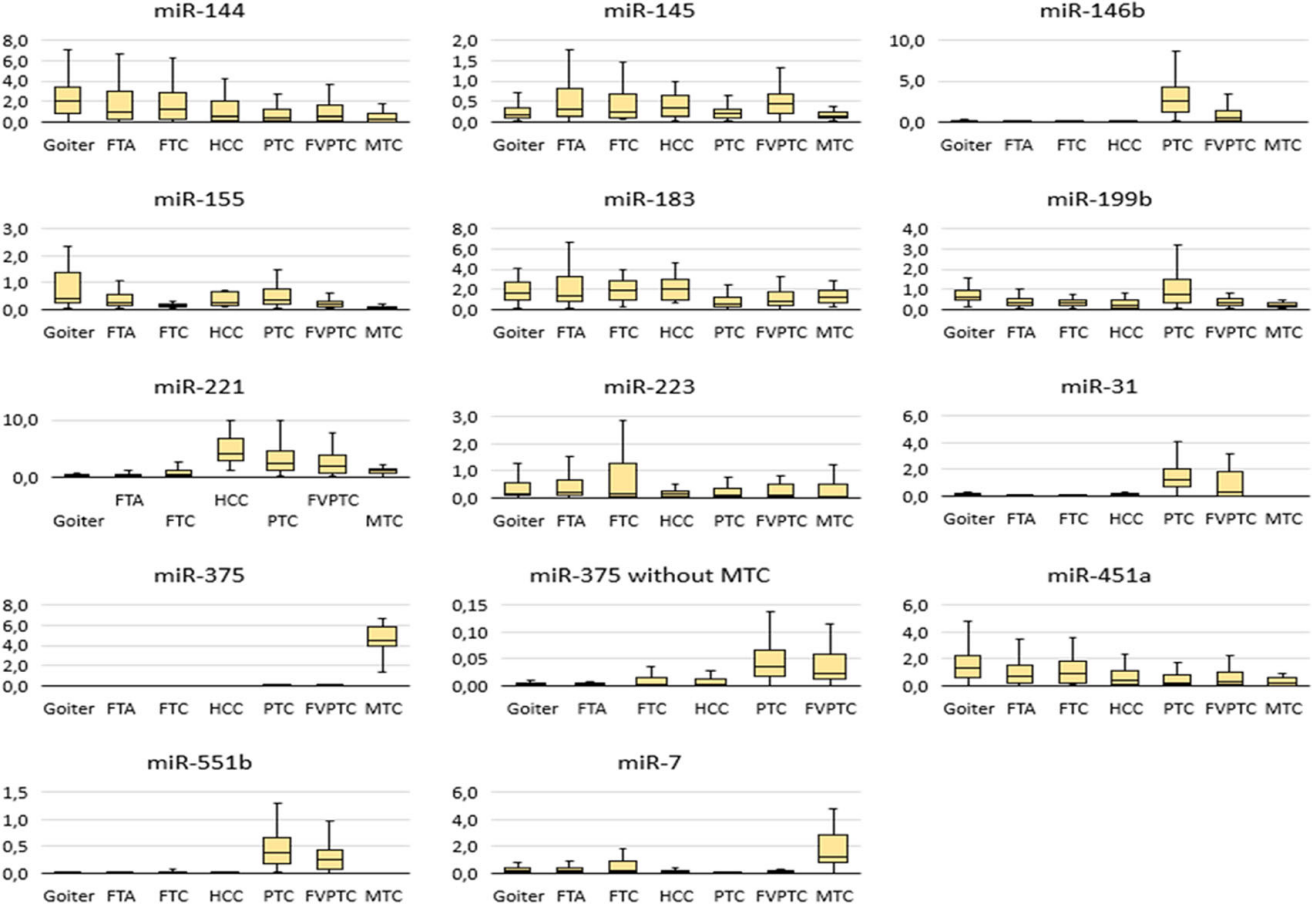
The relative expression level of 13 miRNAs in goiters and different tumor types. The data are normalized; the figure presents the median value, upper and lower quartiles, and nonoutlier range; outliers are not shown

The normalized expression levels of the 13 miRNAs in goiters and various tumor types are depicted in Fig. 4. It is obvious that in PTCs, the expression of miR-146b, − 31, −551b, − 221, and − 375 is significantly higher; by contrast, in MTCs, the levels of miR-146b, − 155, − 31, and -551b are low, while the level of miR-375 is high, to a much greater extent relative to PTCs. In HCCs, only the miR-221 level is significantly high.

For the discrimination of all the samples into benign (including goiter and follicular adenomas) and malignant by means of a single miRNA, the highest ROC AUC values were obtained for three miRNAs: miR-146b (AUC = 0.728 ± 0.045), miR-221 (AUC = 0.872 ± 0.03), and miR-375 (AUC = 0.846 ± 0.036). At the same time, miR-146b was not upregulated in some types of malignant tumor (see Fig. 4) and therefore cannot be considered a universal marker of malignancy. MiR-221 and miR-375 were the most universal markers because their higher expression as compared with benign tumors and goiters was noted in all types of carcinomas. See also Additional file 2: Table S2, for the data on goiters and each tumor type.

### The identification of goiters and tumor histotypes using molecular markers and the resulting problems

Initially for training the classifier, we utilized the data from pathology reports. The first step involved discrimination between goiters and tumors (including follicular adenomas). Total error in this case was 3.25%, total cross-validation error was 12.86%, and the other data are given in Table 2.

**Table 2:** The classifier characteristics for discrimination between goiters and tumors

Characteristics of classifier			Cross-validation	
Group	Sensitivity	PPV	Sensitivity	PPV
Goiters	0.9429	0.9083	0.6871	0.7020
Tumors	0.9742	0.9844	0.9212	0.9160

At the second step, the set of tumors was divided into PTCs, FVPTCs, MTCs, HCCs, and FNs. Total error in this case was 4.38%, total cross-validation error was 16.89%, and the other data are listed in Table 3.

**Table 3:** The classifier characteristics for categorizing tumors as PTC, FVPTC, MTC, HCC, or FN

Classifier characteristic			Cross-validation	
Group	Sensitivity	PPV	Sensitivity	PPV
MTC	0.9474	0.9474	0.7957	0.9024
PTC	0.9421	0.9661	0.8289	0.8401
FVPTC	0.9375	0.9259	0.6889	0.6667
HCC	0.9200	1.0000	0.8160	0.9808
FN	0.9860	0.9592	0.9197	0.8854

The third step was subdivision of the set of FNs into follicular adenomas and carcinomas. Here, total error was 8.39%, total cross-validation error was 24.57%, and the other data are given in Table 4.

**Table 4:** Classifier characteristics for discrimination between FTC and FTA

Classifier characteristic			Cross-validation	
Group	Sensitivity	PPV	Sensitivity	PPV
FTC	0.8605	0.8605	0.5728	0.6010
FTA	0.9400	0.9400	0.8337	0.8169

**Fig. 5:**
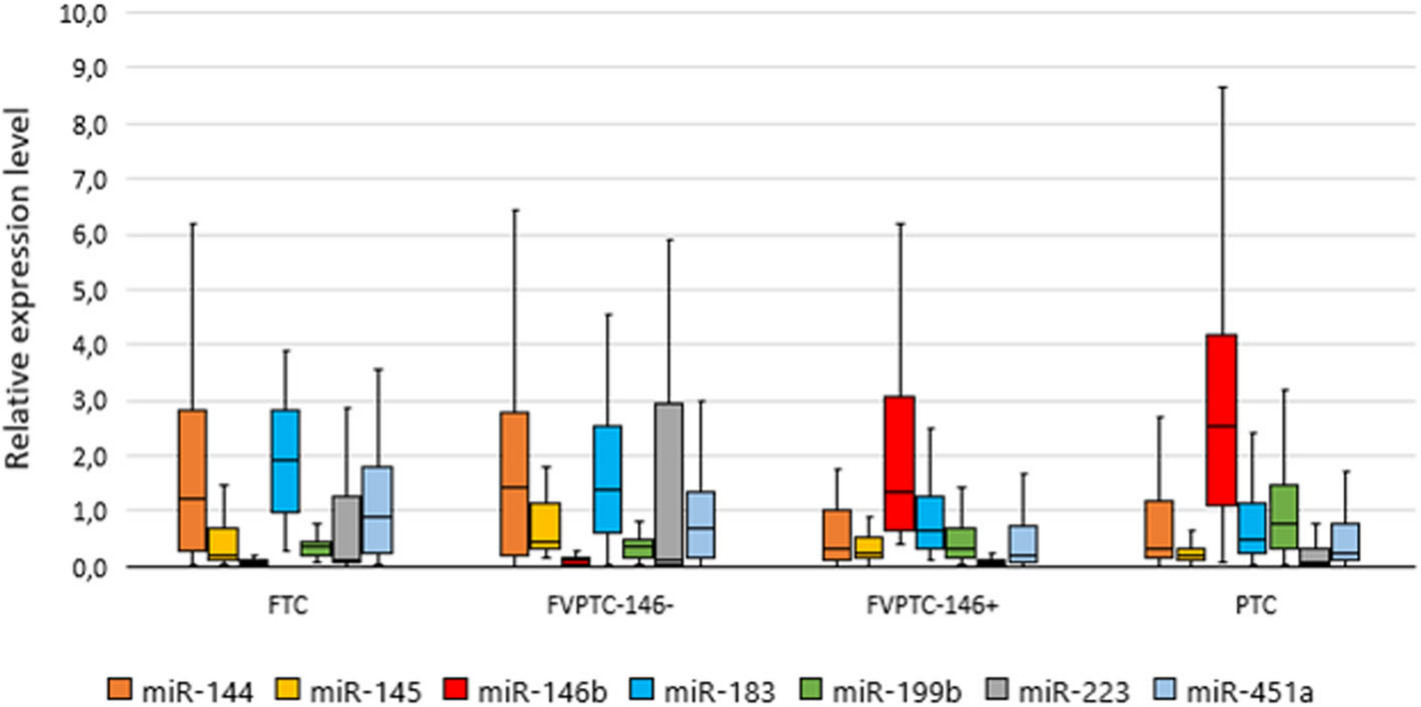
Relative expression levels of miRNAs in FVPTC samples with higher or nonincreased miR-146b levels. The data are normalized; the figure depicts the median value, upper and lower quartiles, and nonoutlier range; outliers are not shown

Thus, during cross-validation, total error at each step increased 3- to 4-fold. In addition, the data in Tables 2, 3, and 4 show that during cross-validation, sensitivity and PPV noticeably dropped for goiters and all tumor types, suggesting their heterogeneity with respect to the levels of selected molecular markers. This was especially true of a) goiters because they differ little from benign follicular adenomas and b) groups FVPTC and FTC. Consequently, the molecular marker profile of some FVPTC samples was similar to that of classic PTC, whereas this profile of some others resembled that of FTC. The most characteristic difference between these subgroups is the presence of miR-146b overexpression typical of classic PTC. It is clear in Fig. 5 that in FVPTCs with overexpressed miR-146b, the changes in the levels of some other miRNAs are also similar to those in classic PTC, whereas in the rest of the samples, they are similar to those in FTC.

Thus, the classification based on molecular markers that we selected does not make it possible to classify thyroid tumors in full accordance with pathology reports. This result seems to be due to the heterogeneity of different histotypes in terms of molecular markers’ levels.

### The molecular-marker–based classification of cytological preparations supports an alternative classification of FNs

**Fig. 6:**
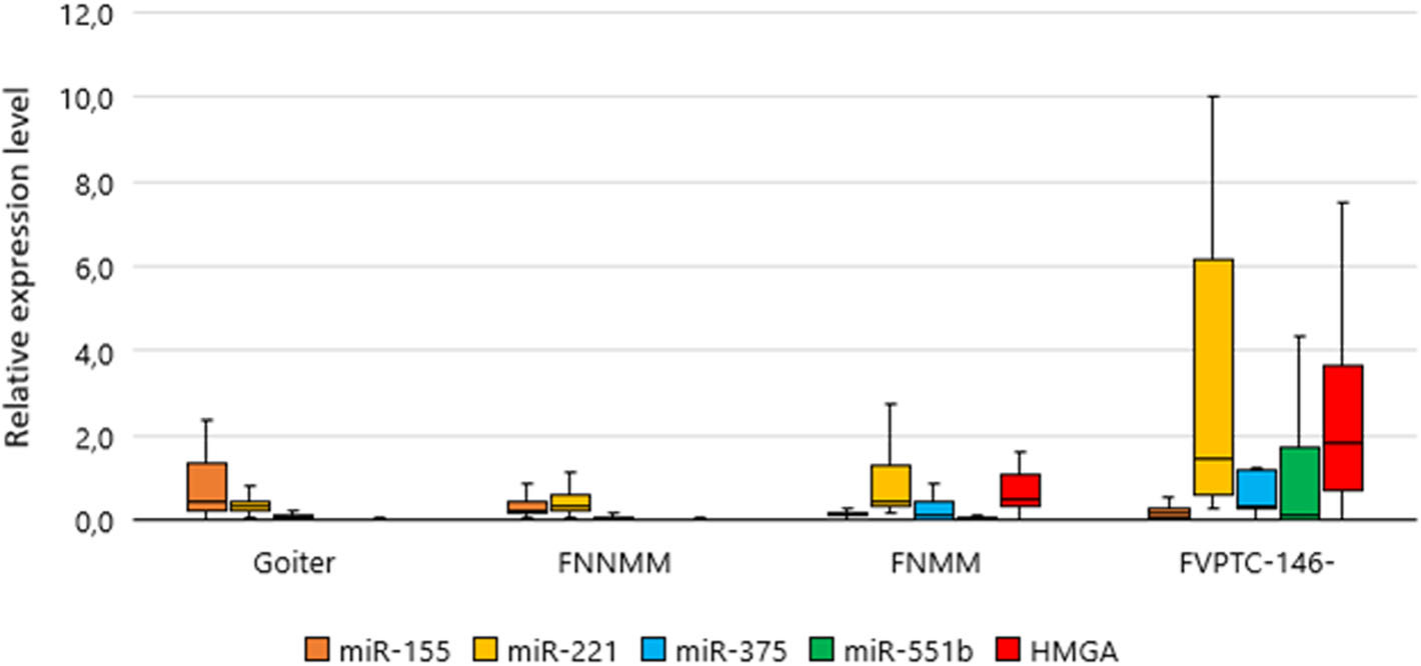
Relative levels of miRNAs and of *HMGA2* expression in groups FNMM and FNNMM in comparison with goiters and the FVPTC subgroup with low expression of miR-146b. The data are normalized; the figure presents the median value, upper and lower quartiles, nonoutlier range, and outliers (circles)

To enhance the robustness of the differential diagnosis of thyroid nodules, we proposed an alternative classification of FNs, which is based not only on morphological characteristics but also on the profile of the molecular markers of malignancy. These changes affected the following two groups:

1. The set of FVPTCs was divided into two groups according to the differences illustrated in Fig. 5. Tumors with overexpressed miR-146b were classified as PTCs (*n* = 43; 54%), and the remaining samples (*n* = 37; 46%) were classified as FNs.
2. The set of FNs, irrespective of the pathology report, was divided into two groups. Tumors with elevated expression of *HMGA2* or miR-221 or miR-375 were classified as “follicular neoplasms with markers of malignancy” (FNMMs), and tumors with the unchanged expression level of all these markers (*HMGA2*, miR-221, miR-375)—typical of goiter—were classified as “follicular neoplasms with no markers of malignancy” (FNNMMs; Fig. 6). In accordance with this stratification, the FNMM group consisted of 73 samples, including 30 FTCs (41%), 37 FVPTCs (51%), and 6 FTAs (8%). The FNNMM group (*n* = 108) was composed of 95 FTAs (88%) and 13 FTCs (12%).

Consequently, all the clinical samples were redistributed into the following groups: benign (goiter+FNNMM), papillary carcinoma, medullary carcinoma, HCC, and FNMMs. For this molecular classification, total error in the discrimination between benign and malignant tumors was 0.81%, total cross-validation error was 1.63%, and the other data are presented in Table 5.

**Table 5:** Classifier characteristics for discrimination between benign and malignant tumors

Classifier characteristic			Cross-validation	
Group	Sensitivity	PPV	Sensitivity	PPV
Benign	0.9953	0.9860	0.9810	0.9810
Malignant	0.9893	0.9964	0.9857	0.9857

When the set of tumors was divided into papillary carcinomas, medullary carcinomas, HCCs, and FNMMs, total error was 1.78%, general cross-validation error was 2.29%, and the other data are presented in Table 6.

**Table 6:** Classifier characteristics for categorization of tumors as PTC, MTC, HCC, or FNMM

Classifier characteristic			Cross-validation	
Group	Sensitivity	PPV	Sensitivity	PPV
MTC	0.9474	1.0000	0.9474	1.0000
PTC	0.9878	0.9878	0.9804	0.9877
HCC	0.9200	1.0000	0.9113	0.9912
FNMM	1.0000	0.9605	1.0000	0.9456

Thus, with the alternative classification, total error, sensitivity, and PPV changed significantly less after cross-validation than when histological classification was chosen as the reference method.

The decision tree for classifying samples into benign and malignant was as follows:

- HMGA2 < 0.0918
  - o miR-375 < − 12.1213
    - ▪ miR-221 < 0.0105
      - ▪ miR-146b < 1.5362 then Diagnosis = Benign (98.6% of 214 examples)
      - ▪ miR-146b ≥ 1.5362 then Diagnosis = Malignant (88.9% of 9 examples)
    - ▪ miR-221 ≥ 0.0105 then Diagnosis = Malignant (100.0% of 19 examples)
  - o miR-375 ≥ − 12.1213 then Diagnosis = Malignant (100.0% of 37 examples)
- HMGA2 ≥ 0.0918 then Diagnosis = Malignant (100.0% of 214 examples)

The minimum set of markers yielding highly accurate discrimination between benign and malignant tumors was an increased expression level of the *HMGA2* gene and of miRNA-375, -221, and -146b (only the markers with the largest AUCs).The decision tree for tumor typing was as follows:

- miR-375 < 5.2514
  - o miR-146b < 0.1721
    - ▪ mtDNA < 5716.3013 then Diagnosis = FNMM (96.0% of 76 examples)
    - ▪ mtDNA ≥5716.3013 then Diagnosis = HCC (100.0% of 23 examples)
  - o miR-146b ≥ 0.1721 then Diagnosis = PTC (98.8% of 164 examples)
- miR-375 ≥ 5.2514 then Diagnosis = MTC (100.0% of 18 examples)

The main characteristic of PTC was an increased level of miR-146b; for MTC, it was an increased level of miRNA-375; the main characteristic of HCC was a high mtDNA/nDNA ratio, and the remaining samples were classified as FNMMs.

**Fig. 7:**
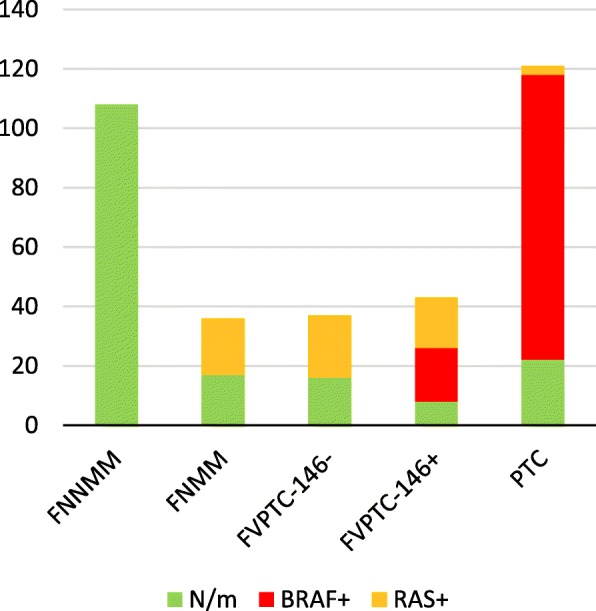
Numbers of samples with *RAS* and *BRAF* mutations from different classes of tumors according to the proposed molecular-marker–based classification

Of note, even though *BRAF* V600E and *RAS* mutations were not included in the decision tree as criteria for the molecular classification of tumors, the distribution of these mutations in the proposed subclasses of FNs turned out to be nonrandom. For example, the *BRAF* V600E mutation was detected exclusively in the FVPTCs classified as PTC by the molecular-marker–based algorithm but in none of the FVPTCs classified as FNMM. Similarly, RAS mutations were detected only in the FN samples included in the group of malignant tumors (Fig. 7).

### Discrepancies between the histological and molecular classifications of thyroid nodules

**Fig. 8:**
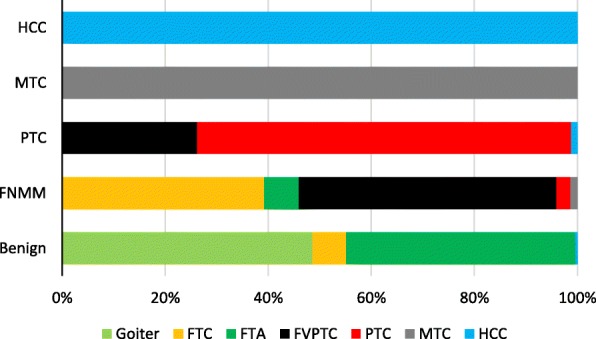
Relative proportions of samples belonging to different classes according to the pathology report but belonging to the same class according to the molecular classifier (indicated on the left): FNMM, follicular neoplasm with markers of malignancy; HCC, Hürthle cell carcinoma; MTC, medullary thyroid carcinoma; PTC, papillary thyroid carcinoma

**Fig. 9:**
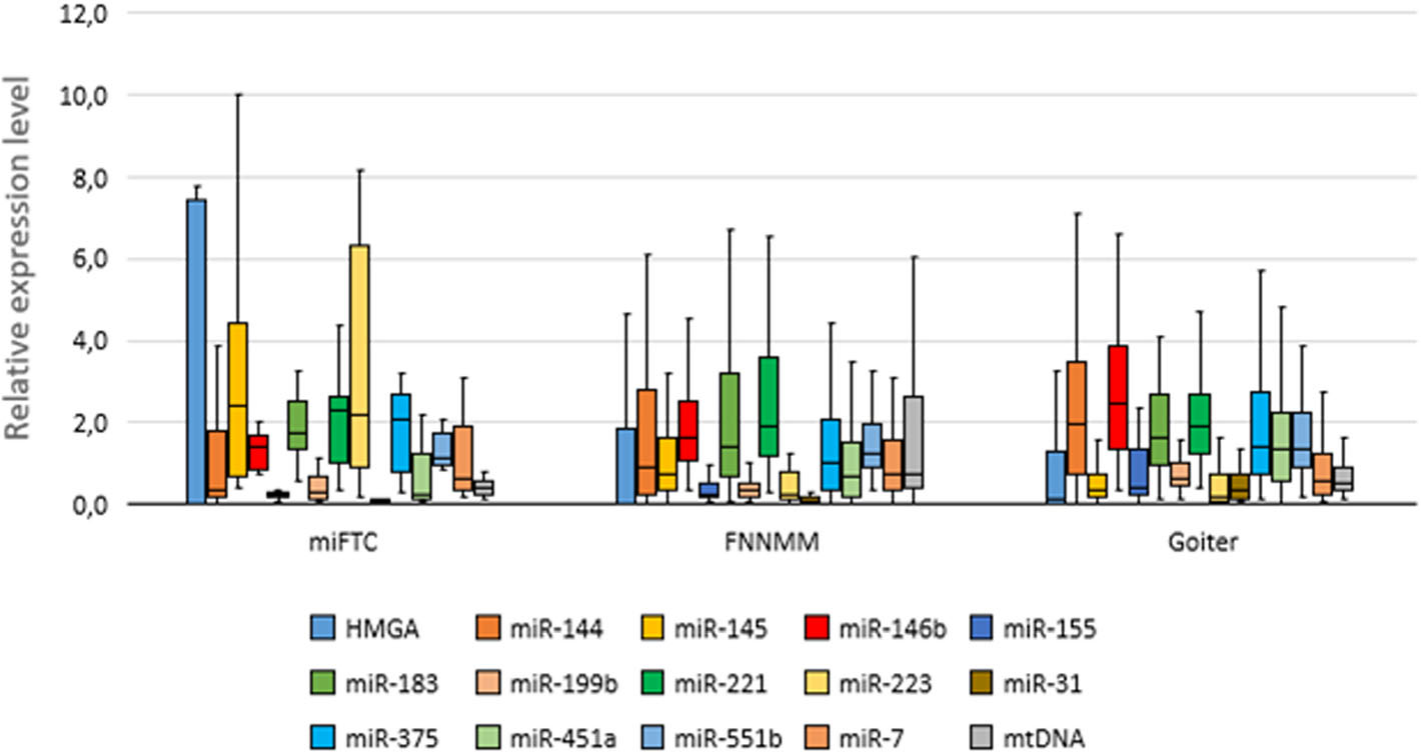
Relative expression levels of miRNAs and *HMGA2* mRNA in minimally invasive FTCs in comparison with FNs without markers of malignancy and goiters. Data are normalized; the figure depicts the median, upper and lower quartiles, nonoutlier range, and outliers (circles)

The structure of matches and discrepancies between these classifications is depicted in Fig. 8. We paid special attention to the situations where the histological classification and molecular classification of a sample led to different conclusions regarding malignancy. Such results were designated as discordant. The situation where a tumor was identified as malignant by the histological analysis but as benign by the molecular classification was assumed to be a false negative result, and the opposite case was termed a false positive. A total of 20 discordant results were obtained (4.04%), of which five (1.01%) were false positive and 15 (3.03%) false negative. In two cases, discordant results were obtained for different preparations from the same patient. In the first case, one of the two preparations with the pathology report “FTA” was identified as FNMM. In the second case, one of the two preparations reported as “HCC” was identified as benign. Most of the false negative results (nine cases) were minimally invasive follicular carcinomas (MIFTCs) that were categorized by the molecular classifier as benign. A detailed analysis of the MIFTC group revealed no significant differences from benign tumors in the profile of most of the selected molecular markers (Fig. 9).

## Discussion

The results suggest that a simple real-time PCR–based analysis of a small set of dissimilar molecular markers (somatic point mutations and translocations and concentrations of specific mRNAs, miRNAs, and mtDNA) can be useful for confirming malignancy. While trying to use a minimum set of markers, we achieved the best combination of sensitivity and specificity by combining markers of two different types. In contrast, a comparable number of markers of the same type (six to eight miRNAs and six to eight somatic mutations and translocations) did not allow us to obtain acceptable results (data not shown), although this solution looked more attractive methodologically. It should be underlined that none of the analyzed somatic mutations and translocations that are widely used as thyroid malignancy markers was selected by our classification algorithm. Meanwhile, the best stand-alone miRNA markers of malignancy (miR-146b, miR-221, and miR-375) as well as the best mRNA marker (*HMGA2* mRNA level) were selected successfully here. Among these, the *HMGA2* mRNA level was found to be the best marker of malignancy not only for the entire sample but also for the heterogeneous group of FNs when considered separately.

Overexpression of genes from the *HMGA* family is typical of malignant tumors and is related to neoplastic cell transformation and rapid tumor progression [31]. The role played by HMGA2 in the formation of malignant tumors of epithelial origin is well known [32, 33]. The association of *HMGA2* mRNA upregulation with malignancy of thyroid tumors has been demonstrated in several publications [15, 16, 34, 35]. In our study, no tumors with overexpressed *HMGA2* were diagnosed as benign in the histological analysis. This result is fairly consistent with our earlier findings where increased HMGA2 expression was not detected in any of 375 specimens cytologically diagnosed as goiters [36].

Despite its high specificity for malignant tumors, HMGA2 upregulation is characteristic only of thyroid carcinoma deriving from follicular cells, not from Hürthle and parafollicular cells; this state of affairs affects its diagnostic sensitivity and NPV when the HMGA2 expression level serves as a molecular marker of malignancy. Accordingly, in ref. [15], real-time RT-PCR measurement of *HMGA2* mRNA levels in cytological preparations, despite fairly high diagnostic specificity (97%), manifested only 71% sensitivity at detecting malignancy with the selected cutoff. In our study, increased *HMGA2* mRNA levels were not observed in some FTCs and PTCs, in most MTCs, and in all HCCs. Nevertheless, the NPV of this marker turned out to be the highest among all the stand-alone markers.

In the cases without *HMGA2* overexpression, the selected panel of three miRNAs resulted in a better combination of sensitivity and specificity for malignancy than did all the selected somatic mutations and translocations taken together. In this study, the *BRAF* V600E mutation and translocations *RET-PTC1* and *PAX8-PPARγ* were very specific for histologically confirmed malignancy; this finding is consistent with published data. On the other hand, their inclusion in the diagnostic panel failed to increase not only diagnostic sensitivity (possibly because of relatively low frequency in the analyzed sample and/or technical constraints) but also diagnostic specificity.*RAS* mutations were significantly more frequent in malignant tumors and were not detected in goiters. Regardless of the classification method, the probability of a tumor with RAS mutations being malignant rather than benign was significantly higher (odds ratio ~ 8–9, data not shown). Nevertheless, given that some RAS mutations have been detected in the tumors histologically classified as follicular adenomas (as reported in other works; see, e.g. [37, 38]), they cannot be regarded as unambiguous signs of malignancy. We believe that these mutations, in contrast to *BRAF* V600E and the translocations, should be considered prognostic rather than diagnostic markers. There is evidence that RAS mutations can point to a reduced risk of aggressive disease if they are found in a malignant tumor [39, 40].

Differential miRNA expression in various thyroid tumor types and at different stages of tumor differentiation or progression has been described in many studies [9, 12, 21, 22, 41–43]. The relevance of miRNA quantitation in cytological samples to preoperative diagnostics has been reported, and diagnostic solutions have been proposed [44–47]. The main constraints associated with the detection of malignancy by miRNA profiling described in numerous works are related to the group of FNs. In this regard, our results are in full agreement with the observations in the above publications. We registered significant changes in the expression of miRNAs characteristic of PTCs and MTCs, which clearly distinguished these malignant tumors from all benign lesions. By contrast, our miRNA panel did not succeed in the precise discrimination of histologically confirmed FTAs from FTCs in terms of malignancy despite the statistically significant differences in the expression levels of miR-31, -145, -155, -199b, and -375 between these groups.

Our results show that our approach can be useful not only for confirming malignancy but also for tumor typing by means of almost the same set of markers. Because miR-221 overexpression was typical of all the malignant tumor types, its quantification appears to be unsuitable for tumor typing. The combination of the other markers reliably distinguished PTCs and MTCs from other diagnoses. As an additional component of the marker panel, we suggested the mtDNA/nDNA ratio to identify HCCs as a separate class, and indeed, this ratio was high in all the samples of this group. Nonetheless, samples with high mtDNA content were also detected among the PTCs and some benign tumors, namely FTAs and goiters. These results may mean Hürthle cell variants of PTC, Hürthle cell variants of FTA, and Hashimoto’s thyroiditis, respectively. Nevertheless, no overexpression of the selected miRNAs or *HMGA2* was detected in any benign tumor or goiter with an elevated mtDNA/nDNA ratio. Thus, by adding this marker to the miRNA panel, we were able to detect HCCs.

According to the profiles of the molecular markers, we divided our sample into five groups. The first group included all goiters, the majority of FTAs, and a minority of FTCs. No suspected markers of malignancy were found in the specimens of this group, and therefore we designated it as “benign.” The second group called “papillary carcinoma” included all classic PTCs and some FVPTCs. The third group included only FNs (most FTCs, some FVPTCs, and some FTAs), where the suspected markers of malignancy were found. The fourth and fifth groups corresponded to the histological diagnoses “medullary carcinoma” and “HCC,” respectively. This reclassification after cross-validation led to a much less significant change in total error, sensitivity, and PPV relative to the classification based on histology reports. This finding points to much greater homogeneity of the groups in terms of distribution of the molecular markers from the proposed panel.

Our results are fully consistent with recent studies on classification of thyroid tumors by molecular methods. Virtually every study in which such an attempt was made has encountered difficulties with separating FNs consistently with the histological classification (FTC or FTA), regardless of the nature or number of the molecular markers involved. The evidence accumulated in recent years indicates that the profile of molecular markers for certain types of thyroid tumors is not consistent with the histological classification.

First, this is true of FVPTCs, which can be categorized into subclasses with markedly different morphological features, sets of characteristic mutations, and gene expression profiles. Accordingly, in some studies [48, 49], it was shown that infiltrative FVPTCs are characterized by a higher incidence of the BRAF V600E mutation and *RET-PTC* translocations but a lower frequency of RAS mutations in comparison with encapsulated FVPTC. Conversely, the BRAF V600E mutation and the *RET-PTC* translocation are uncharacteristic of encapsulated FVPTC. In a study by Yoo and coworkers [49], comprehensive molecular profiling of a sample containing 180 thyroid tumors of different types enabled their subdivision into three classes: BRAF-like, RAS-like, and nonBRAF-nonRAS. In that study, encapsulated versions of FVPTC almost exclusively fell into to the RAS-like group with a high frequency of RAS mutations in the absence of BRAF mutations. The same group also included a significant proportion of follicular carcinomas. Most of infiltrative FVPTCs, in turn, fell into the BRAF-like group, like almost all classic PTCs, regardless of the presence of the BRAF V600E mutation. Furthermore, there were no follicular carcinomas in this group. In ref. [12], a wide range of molecular markers was comprehensively analyzed, including miRNAs, mRNAs, somatic mutations and translocations, copy number variations, and methylation patterns in more than 400 papillary carcinomas. FVPTCs clustered into two groups with different mutation patterns and gene expression profiles: BRAF-like and RAS-like, with the BRAF-like group being characterized by higher miR-146b content in comparison with the RAS-like group. Our data are in line with the results of the above-mentioned reports because all the specimens histologically diagnosed as FVPTC were redistributed either to the PTC group with a high frequency of the *BRAF* V600E mutation and a relatively high miR-146b level or to the FNMM group without the *BRAF* V600E mutation but with relatively low miR-146b content.

Second, FNs (Bethesda category IV) are fairly well distinguishable from the other tumor types but cannot be clearly subdivided into FTCs and FTAs by molecular typing. Accumulating evidence suggests that this category of tumors does not represent two discrete classes with respect to the levels of many molecular markers and metabolites but rather represents a range of transitional types [35, 50]. Therefore, in some nodules, molecular markers of malignancy can be detected even before morphological changes take place. Hence, the conclusion about malignancy based on molecular typing may differ from that in the pathology report. Such cases can be considered a false positive result of molecular analysis but actually reflect a difference in the predictive value of the two types of test. Interpretation of such molecular-typing results for disease management may require further research, including follow-up, where molecular data will be compared not only with histological but also with clinical data. For example, MIFTCs, which accounted for the majority of “false negative” results of our molecular typing, are considered in some studies to be indolent, similar in clinical outcomes to follicular adenomas, and requiring more sparing measures for disease management than widely invasive FTC does [51, 52]. Several publications suggest that the gene expression patterns of MIFTCs are much closer to those of FTAs than of widely invasive FTCs [49, 52, 53].

Our study has several limitations: e.g., we analyzed only the samples that belong to the II, IV, and VI Bethesda categories, as predetermined by the design of the study; some types of thyroid tumors (for example, noninvasive follicular thyroid neoplasms with papillary-like nuclear features or papillary microcarcinomas) were not included in the sample because they were not available. Further expanded studies including an analysis of specimens from other Bethesda categories (I, III, and V) are necessary to identify the drawbacks of the proposed panel and for possible improvements.

## Conclusions

Our study indicates the feasibility of detecting and typing malignant thyroid tumors using a small panel of molecular markers detected in cytological preparations by a simple PCR-based method. Combining markers of different types made it possible to achieve high PPV at acceptable NPV. It should be emphasized that the proposed panel did not yield any false negative results during the testing of goiters and papillary cancers. Taking into account that more than 90% of thyroid nodules are benign (of which ~80% are goiters) and that the vast majority (~80–90%) of the malignant tumors are PTCs, rather high NPV can be expected when the proposed approach is used for routine tests at healthcare institutions. The method is potentially integrable into existing disease management protocols because it does not require separate sampling and nucleic-acid extraction. It can be applied in combination with cytological analysis and thereby can help reduce the number of additional biopsies and unnecessary surgical interventions.

## Supplementary information

**Additional file 1: Table S1.** The oligonucleotide sequences used in the study.

**Additional file 2: Table S2.** Relative expression levels of *HMGA2* and of miRNAs and the mtDNA/nDNA ratio in goiters and various tumor types. Blank cells denote missing data. FTA, follicular thyroid adenoma; FTC, follicular thyroid carcinoma; FVPTC, follicular variant of papillary thyroid carcinoma; HCC, Hürthle cell carcinoma; MTC, medullary thyroid carcinoma; PTC, papillary thyroid carcinoma. BRAF “1”: the V600E mutation detected; RAS “1”: one of the following mutations detected: Q61R in the *HRAS* gene or Q61K, Q61R, or Q61L in *NRAS*.

## Data Availability

All data generated or analyzed during this study are included in this published article and its supplementary information files.

## Abbreviations

AUC: Area under the ROC curve
FN: Follicular neoplasm
FNA: Fine-needle aspiration
FNAC: Fine-needle aspiration cytology
FNMM: Follicular neoplasm with markers of malignancy
FNNMM: Follicular neoplasm with no markers of malignancy
FTA: Follicular thyroid adenoma
FTC: Follicular thyroid carcinoma
FVPTC: Follicular variant of papillary thyroid carcinoma
HCC: Hürthle cell carcinoma
MIFTC: Minimally invasive follicular carcinoma
MTC: Medullary thyroid carcinoma
NPV: Negative predictive value
PPV: Positive predictive value
PTC: Papillary thyroid carcinoma
ROC: Receiver-operating characteristic
